# The protective role of l-carnitine on oxidative stress, neurotransmitter perturbations, astrogliosis, and apoptosis induced by thiamethoxam in the brains of male rats

**DOI:** 10.1007/s00210-023-02887-7

**Published:** 2023-12-15

**Authors:** Heba-Tallah Abd Elrahim Abd Elkader, Marium Marzoq Hussein, Nema A . Mohammed, Heba M . Abdou

**Affiliations:** 1 Zoology, Biological and Geological Sciences Department, Faculty of Education, Alexandria University, Alexandria, Egypt; 2 Zoology Department, Faculty of Science, Omar Al-Mukhtar University, Al-Bayda, Libya; 3 Zoology Department, Faculty of Science, Alexandria University, Alexandria, Egypt

**Keywords:** Thiamethoxam, l-Carnitine, Wistar rats, Oxidative stress, Astrogliosis, Apoptosis

## Abstract

Synthetic organic insecticides such as pyrethroids, organophosphates, neonicotinoids, and others have the potential to disrupt ecosystems and are often toxic to humans. Thiamethoxam (TMX), a neonicotinoid insecticide , is a widely used insecticide with neurotoxic potential. l-Carnitine (LC) is regarded as the “gatekeeper” in charge of allowing long-chain fatty acids into cell mitochondria. LC is an endogenous chemical that is renowned for its prospective biological activity in addition to its role in energy metabolism. This study investigated the protective effects of LC against TMX-induced neurotoxicity in male Wistar rats. For 28 days, animals were divided into four groups and treated daily with either LC (300 mg/kg), TMX (100 mg/kg), or both at the aforementioned doses. Our results revealed marked serum lipid profile and electrolyte changes, declines in brain antioxidants and neurotransmitters (acetylcholine, dopamine, and serotonin levels) with elevations in thiobarbituric acid reactive substances and proinflammatory cytokine levels, as well as acetylcholinesterase and monoamine oxidase brain activity in TMX-treated rats. TMX also increased the expression of caspase-3 and glial fibrillary acidic protein. In contrast, pretreatment with LC attenuated TMX-induced brain injury by suppressing oxidative stress and proinflammatory cytokines and modulating neurotransmitter levels. It also ameliorated the expression of apoptotic and astrogliosis markers. It could be concluded that LC has antioxidant, anti-inflammatory, anti-astrogliosis, and anti-apoptotic potential against TMX neurotoxicity.

**Graphical abstract:**

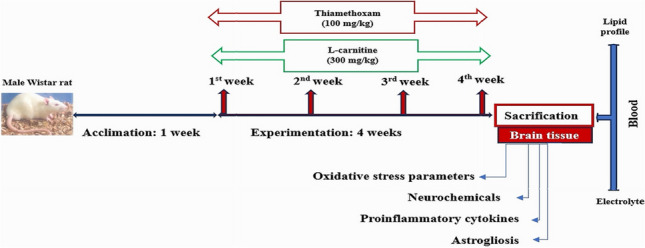

## Introduction

Neonicotinoids (NEOs), a class of acetylcholine receptor inhibitors, are the most widely used pesticides, accounting for 30% of the global insecticide market and being registered in over 120 countries worldwide (Li et al. 2022). Because of their advantages, such as high efficacy against sucking insects, ease of use, and low cost, they have gradually replaced organophosphorus, pyrethroid, and carbamate insecticides (Distefano et al. 2022). NEOs are made up of seven compounds that are classified into three generations: the first (imidacloprid, nitenpyram, acetamiprid, and thiacloprid), the second (thiamethoxam and clothianidin), and the third (dinotefuran) (Li et al. 2022). With the increased use of NEOs, they have been detected in a variety of environmental matrices, including soil (Chen et al. 2022), water, and food (Naumann et al. 2022), and have been linked to adverse health effects in nontarget organisms (Zhang et al. 2021; Addy-Orduna et al. 2022). Despite the initial belief that NEOs have low mammalian toxicity, there is growing evidence that NEOs can cause a wide range of toxic effects in animals and humans, including neurotoxicity, immunotoxicity, hepatotoxicity, nephrotoxicity, and reproductive cytotoxicity in vertebrates and invertebrates (Wang et al. 2018; Anadón et al. 2020). They effectively block acetylcholine (Ach) binding and overstimulate cells at synaptic junctions, obstructing nerve impulse propagation and leading to cell and/or individual paralysis and death (Almeida et al. 2021).

Thiamethoxam (TMX; 3-(2-chloro-1,3-thiazol-5-ylmethyl)-5-methyl-1,3,5-oxadiazine-4-ylidene (nitro) amine) is a representative second-generation neonicotinoid (NEO) insecticide with a broad activity spectrum and high target specificity against different types of insects, as well as a low risk to nontarget mammalian species (Liu et al. 2018). TMX is a WHO class III carcinogen with the liver and kidney as its primary targets, and lifelong-fed mice have an increased incidence of liver tumors (Yi et al. 2023). TMX, as an agonist of nicotinic acetylcholine receptors (nAChRs) in the nervous system, can cause neurological disorders and systemic neurotoxicity by disrupting synaptic transmission in the nervous system (Yang et al. 2023). TMX can also bind to Ach receptors and reduce Ach-induced action potentials, causing changes in cholinergic-related behavioral and biochemical processes as well as increased anxiety in rats (Yi et al. 2023).

l-Carnitine (LC; β-hydroxy-γ-trimethyl-amino-butyric acid) is a water-soluble antioxidant found in the liver, kidney, and brain of most mammals (El-Sherbini et al. 2017; Nouri et al. 2022). LC is derived from various foods (75%), and the body synthesizes it using various essential amino acids (25%), such as lysine and methionine (Hamza et al. 2020). The biologically active enantiomer LC mediates the transport of long-chain fatty acids into the mitochondrial matrix for cellular energy metabolism and has antioxidant and anti-inflammatory properties (Abdulidha et al. 2020; Sarzi-Puttini et al. 2021). LC is also important in cell osmoregulation and in the stabilisation of cellular and mitochondrial membranes, thereby preventing cell damage (Türkyılmaz et al., 2010). Because LC is easily transported through the blood˗brain barrier via the organic cation/carnitine transporter novel family member 2 (OCTN2), its plasma and cerebrospinal fluid concentrations increase after oral administration (Sarzi-Puttini et al. 2021). Furthermore, LC protects against neurotoxicity and decreases Ach activity and thus cognitive abilities (Mahmoud et al. 2021). This was the first study to investigate the effect of LC on TMX-induced neurotoxicity in male rats. The current study aimed to investigate the effects of LC on oxidative stress, inflammatory factors, and neurochemicals, and the expression of regulatory astrogliosis and apoptosis markers induced by TMX in male Wistar rat brains.

## Materials and methods

### Chemicals

A 25% TMX (Actara®, Syngenta Canada Inc.) was purchased from a local pesticide market. LC was purchased from an Arab company for Pharmaceuticals and Medical Plants (Egypt). All other chemicals used in our experiment were of analytical grade.

### Animal care

Wistar male rats weighing 180 ± 20 g were obtained from the animal house of the Medical Research Institute, Alexandria University. The rats were group-housed in plastic cages in normal laboratory conditions regarding humidity at a temperature of 28 ± 3 °C and a 12-h light/12-h dark cycle, fed a standard pellet diet, and provided drinking water ad libitum. All animals were accommodated in laboratory conditions for 1 week before treatment and maintained under the same conditions throughout the experiment. The study protocol was approved by the Ethical Committee for the Use and Care of Laboratory Animals established by Alexandria University (Alexandria, Egypt). Animal experiments received approval from the Ethical Committee (No. AU 04230427301).

### Experimental design

Twenty rats were randomly divided into four groups with five animals in each group as follows: control animals were administered distilled water as a vehicle, LC (300 mg/kg), TMX (100 mg/kg; 1/15.6 from LD50), and LC + TMX. LC was administered first, and after 30 min, TMX was administered. The doses of TMX and LC were chosen according to previous studies (Feki et al. 2019; Essawy et al. 2022, respectively). The rats were treated orally for 4 weeks daily.

###  Blood and brain tissue preparation

At the end of the study period, all rats were sacrificed by decapitation, under ketamine (100 mg/kg) and xylazine (20 mg/kg) anesthesia, and blood was withdrawn by intracardiac puncture. The serum was immediately separated by centrifugation at 3000 rpm for 15 min at 4 °C and stored at −80 °C. The whole brain of each animal was rapidly dissected, thoroughly washed with ice-cold isotonic saline, dried, weighed, and then divided into two portions. The first portion was directly homogenized in ice-cold 10 mM phosphate buffer (pH 7.4) to prepare a 10% (w/v) homogenate, which was centrifuged at 5000 rpm for 10 min at 4 °C. The supernatant was collected and stored at −80 °C and then utilized for biochemical analyses. The second portion was frozen in liquid nitrogen and stored at −80 °C for RNA extraction.

### Blood lipid profile and electrolyte assays

Serum samples were assayed for lipid profiles (triglycerides (TG), total cholesterol (TC), high-density lipoprotein cholesterol (HDL-C), and low-density lipoprotein cholesterol (LDL-C)) using colorimetric kits from Bio-System Company (Egypt).

Serum electrolyte levels (Na^+^ and Ca^+2^) were determined by an automatic electrolyte analyzer (PL1000A).

### Determination of oxidant/antioxidant capacity biomarkers

Lipid peroxidation was measured using the thiobarbituric acid reactive substances (TBARS) assay by Tappel and Zalkin (1959). The TBARS concentration was calculated using standard curves of increasing 1,1,3,3-tetramethoxypropane concentrations and expressed as nmol/g tissue.

In addition, the levels of reduced glutathione (GSH) as a nonenzymatic antioxidant biomarker were determined based on the method of Jollow et al. (1974). Briefly, the supernatant was centrifuged with 5% trichloroacetic acid. To 0.1 ml of homogenate, 2 ml of phosphate buffer (pH 8.4), 0.5 ml of dithiobis (2-nitrobenzoic acid) (DTNB), and 0.4 ml of double distilled water were added, and the absorbance was read at 412 nm. The results were expressed as mg/g tissue.

Furthermore, three enzymatic antioxidants, superoxide dismutase (SOD), catalase (CAT), and glutathione peroxidase (GPx), were evaluated according to the methods of Mishra and Fridovich (1972), Aebi (1984), and Flohé and Günzler (1984), respectively.

SOD activity was assayed by the inhibition of epinephrine auto-oxidation in an alkaline medium (pH 10.2) to adrenochrome, which is markedly inhibited by the presence of SOD. Epinephrine was added to the assay mixture, containing tissue supernatant, and the change in extinction coefficient was followed at 480 nm in a spectrophotometer. The enzyme activity was expressed as U/g.

CAT activity in brain supernatants was determined by the decomposition of hydrogen peroxide according to Aebi (1984) using a reaction mixture consisting of hydrogen peroxide (H_2_O_2_; 10 mmol/l final concentration) in phosphate buffer (pH 7.0) as the substrate. Changes in absorbance due to H_2_O_2_ degradation were monitored spectrophotometrically at 240 nm for 1 min, and the enzyme activity was expressed as U/g tissue.

GPx activity was measured using cumene hydroperoxide as a substrate. The assay method is based on monitoring the generation of GSH from glutathione disulfide (GSSG) by the action of glutathione reductase in the presence of reduced nicotinamide adenine dinucleotide phosphate (NADPH). The absorbance at 340 nm was recorded. The enzyme activity was expressed as mU/g tissue.

### Determination of inflammatory markers

To evaluate neuroinflammation in brain tissue treated with TMX and/or LC, proinflammatory markers such as tumor necrosis factor-α (TNF-α), interleukin-6 (IL-6), interleukin-1β (IL-1β), and nuclear factor kappa B (NF-κB) were measured by enzyme-linked immunosorbent assay (ELISA) kits obtained from ABclonal and Biosourse (USA), respectively, according to the manufacturer’s instructions for TNF-α (ABclonal, cat no. RK00029), IL-6 (Biosourse, cat no. MBS726707), IL-1β (Biosourse, cat no. MBS825017), and NF-κB (Biosourse, cat no. MBS453975).

### Neurochemical biomarkers

Acetylcholinesterase (AchE) activity was assessed by using commercial rat ELISA kits obtained from Elabscience (cat no. E-EL-R0355) according to the manufacturer’s instructions. Enzyme activity was determined using a molar extinction coefficient of 412 nm.

The activity of monoamine oxidase (MAO) was estimated using p-tyramine hydrochloride as a substrate according to the method of Sandler et al. (1981).

The levels of Ach (cat no. CEA912Ge), dopamine (DA, cat no. DOP31-K01), and serotonin (5-HT, cat no. CSB-E08364r) were estimated in the brain by using commercial rat ELISA kits obtained from Cloud-Clone Crop, Eagle Biosciences Inc, and Cusabio (USA), respectively. The optical density was read at 450 nm in a microplate photometer within 15 min according to the manufacturer’s protocol.

### Quantitative real-time polymerase chain reaction (qRT˗PCR)

Total RNA was extracted from 30 mg of brain tissue samples using TRIzol reagent (Invitrogen, cat no. 15596-026). The extracted RNA concentration was quantified using NanoDrop spectrophotometry (Thermo Fisher Scientific, USA); then, 110 ng of total RNA was transcribed using RNA reverse transcriptase kits (cat no. K0251, Thermo Fisher Scientific, USA). The thermal cycler was programmed at 25 °C for 10 min, 37 °C for 120 min, 85 °C for 5 min, and 4 °C for 20 h. Prepared cDNA was used in the qPCR analyzer (StepOne, Applied Biosystems, Singapore) using MAXIMA SYBR Green qPCR Master Mix with the following program: 1 cycle at 95 °C for 10 min; 40 cycles of 95 °C for 15 s, 60 °C for 30 s and 72 °C for 30 s; and one cycle at 95 °C for 15 s, 60 °C for 1 min and 95 °C for 15 s. The primer sequences used and the sizes of caspase-3 and glial fibrillary acidic protein (GFAP) housekeeping β-actin are shown in Table 1. Duplicate plates were tested for each condition and were compared to assess the reproducibility of the results. The threshold cycle (Ct) for each well was recorded, and data analysis was performed by the 2^−ΔΔCT^ method with normalization to β-actin expression.

**Table 1:** Primer sequences specific for the analyzed genes

Name	Size (bp)	Accession no.	Forward	Reverse
β-Actin	81	NC_051347.1	ATGTGGCTGAGGACTTTGATT	ATCTATGCCGTGGATACTTGG
Caspase-3	160	NC_051351.1	CTTGGAACGCGAAGAAAAGT	AGCCCATTTCAGGGTAATCC
GFAP	121	NC_051345.1	GAAGAAAACCGCATCACCAT	CCGTCTTTACCACGATGTTC

### Statistical analysis

Values are expressed as the means ± SE. The normality of data distribution was assessed using Shapiro-Wilk’s test. Significant differences between values were analyzed by two-way analysis of variance (ANOVA) followed by post hoc Tukey’s multiple comparisons tests. *P* values < 0.05 were considered statistically significant. The study was carried out using GraphPad Prism 6.0 Software (USA).

## Results

### Ameliorative effect of LC on TMX-induced changes in the serum lipid profile and electrolyte ions

**Fig. 1:**
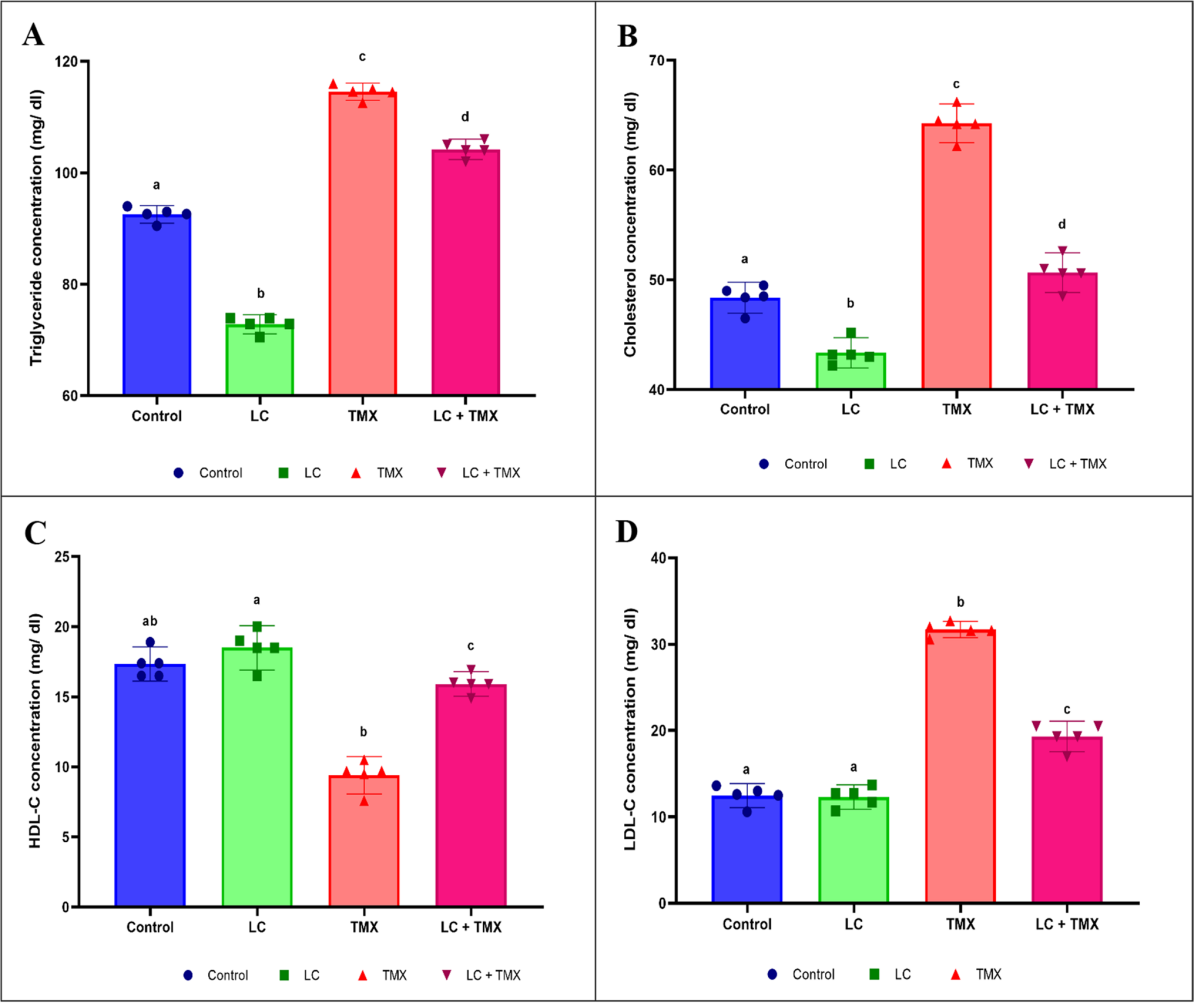
Effect of l-carnitine (LC) on the levels of (**A**) triglycerides (TG), (**B**) total cholesterol (TC), (**C**) high-density lipoprotein cholesterol (HDL-C), and (**D**) low-density lipoprotein cholesterol (LDL-C) in the serum of rats treated with thiamethoxam (TMX). Values are presented as mean ± S.E.; *n* = 5 animals; different superscripts on the columns are significantly different at *P <* 0.05. a, *P* < 0.05 vs. control. b, *P* < 0.05 vs. LC. c, *P* < 0.05 vs. TMX. d, *P* < 0.05 vs. LC + TMX

**Fig. 2:**
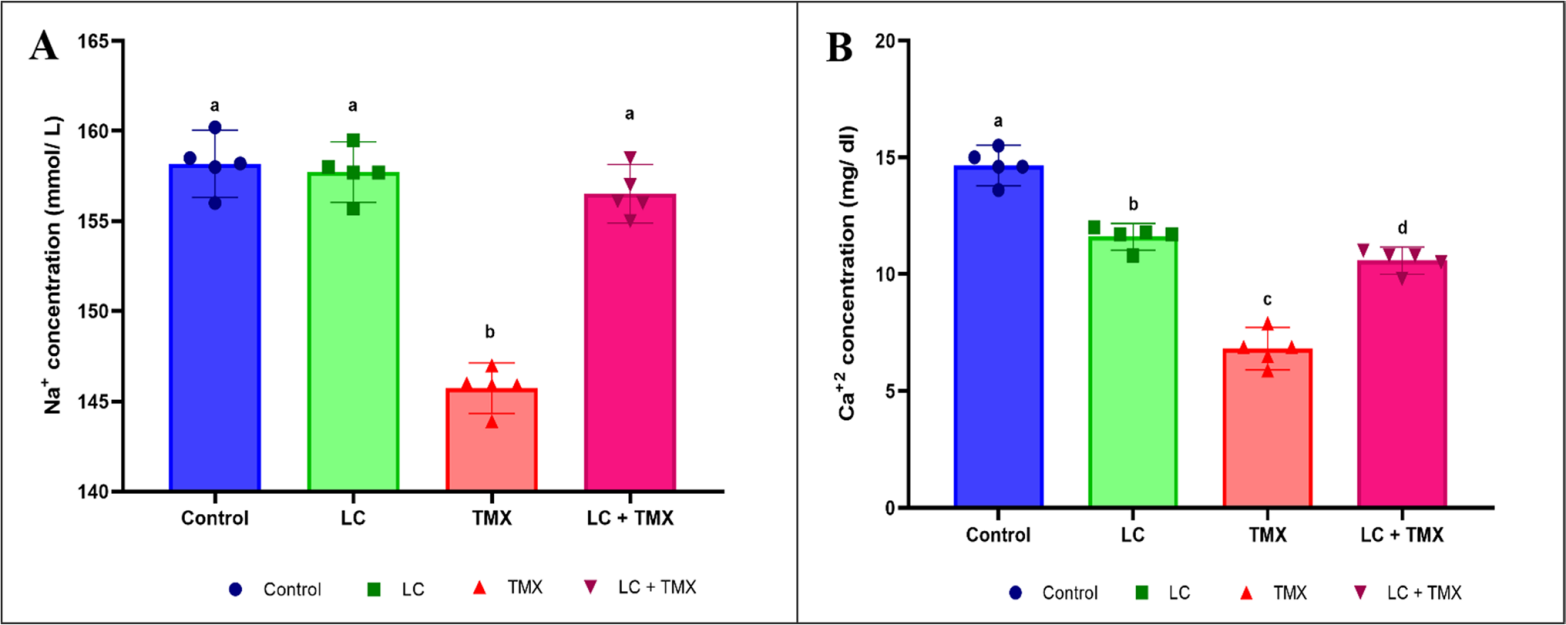
Effect of l-carnitine (LC) on the levels of (**A**) Na^+^ and (**B**) Ca^+2^ in the serum of rats treated with thiamethoxam (TMX). Values are presented as mean ± S.E.; *n* = 5 animals; different superscripts on the columns are significantly different at *P <* 0.05. a, *P* < 0.05 vs. control. b, *P* < 0.05 vs. LC. *c*, *P* < 0.05 vs. TMX. d, *P* < 0.05 vs. LC + TMX

Serum levels of TG, TC, and LDL-C were significantly (*P* < 0.05 vs. controls; 57, 48, and 158%, respectively) higher in TMX-treated rats, while HDL-C was significantly lower (Fig. 1A–D; −49%). Similarly, TMX-treated animals had significantly lower ionic Na^+^ (−8%) and Ca^+2^ (−41%) levels than the control group (Fig. 2A, B). Oral administration of LC and TMX, on the other hand, resulted in significant (*P* < 0.05 vs. TMX group) decreases in TG, TC, LDL-C, and elevated HDL-C levels (Fig. 1A–D; −9, −21, −39, and 69%, respectively). Furthermore, when compared to the TMX-treated group, administration of LC + TMX resulted in a significant improvement in ionic electrolyte levels. Two-way ANOVA revealed a significant interactive effect on TG (*F* = 1.012; *P* = 0.001), TC (*F* = 3.196; *P* = 0.001), LDL-C (*F* = 5.532; *P* = 0.001), and HDL-C (*F* = 4.401; *P* = 0.001) levels. Furthermore, when compared to the TMX-treated group, administration of LC + TMX resulted in a significant improvement in ionic electrolyte (*F* Na^+^ = 2.701, *P* = 0.001; *F* Ca^+2^ = 6.542, *P* = 0.001) levels.

### Ameliorative effect of LC on TMX-induced oxidant/antioxidant imbalance

**Fig. 3:**
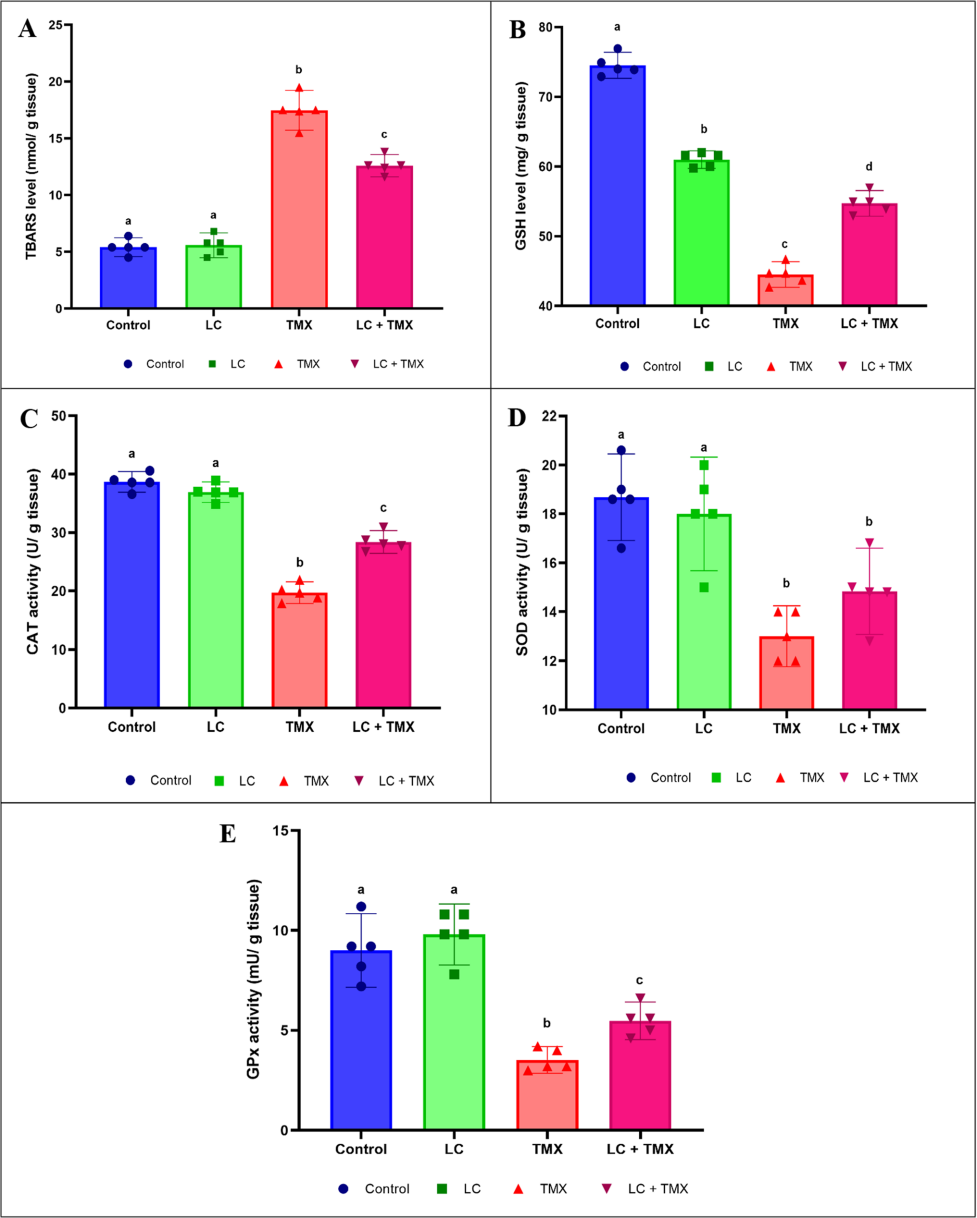
Effect of l-carnitine (LC) on the levels of (**A**) thiobarbituric acid reactive substances (TBARS) and (**B**) reduced glutathione (GSH) as well as the activities of (**C**) catalase (CAT), (**D**) superoxide dismutase (SOD), and (E) glutathione peroxidase (GPx) in the brain of rats treated with thiamethoxam (TMX). Values are presented as mean ± S.E.; *n* = 5 animals; different superscripts on the columns are significantly different at *P <* 0.05. a, *P* < 0.05 vs. control. b, *P* < 0.05 vs. LC. c, *P* < 0.05 vs. TMX. d, *P* < 0.05 vs. LC + TMX

Figure 3A and B depicts the effect of LC administration on TMX-induced oxidative stress. TMX significantly (*P* ˂ 0.05 vs. controls) increased the levels of TBARS (*F* (3, 16) = 178.42; *P* < 0.001; 213%) while decreasing the levels of GSH (*F* (3, 16) = 409.06; *P* < 0.0001; −27%) in the exposed rat brains. Coadministration of LC and TMX significantly improved the levels of TBARS and GSH (−28 and 23%, respectively) in the brain compared to animals exposed to TMX alone. Two-way ANOVA revealed a significant interactive effect of LC and TMX on TBARS (*F* = 2.194; *P* = 0.001) and GSH (*F* = 3.572; *P* = 0.001) levels.

TMX-treated rats had significantly lower activities of CAT (*F* (3, 16) = 137.80; *P* < 0.001; −47%), SOD (*F* (3, 16) = 16.75; *P* < 0.0001; −28%), and GPx (*F* (3, 16) = 28.86; *P* < 0.0001; −64%). The significant decreases in antioxidant status (44, 14, and 56%, respectively) caused by the coadministration of LC and TMX were increased when compared to rats treated with TMX (Fig. 3C–E). Two-way ANOVA revealed a significant interactive effect of LC and TMX on CAT (*F* = 8.751; *P* = 0.001), SOD (*F* = 2.437; *P* = 0.0001), and GPx (*F* = 637.236; *P* = 0.0001).

### Ameliorative effect of LC on TMX-induced inflammatory stress

**Fig. 4:**
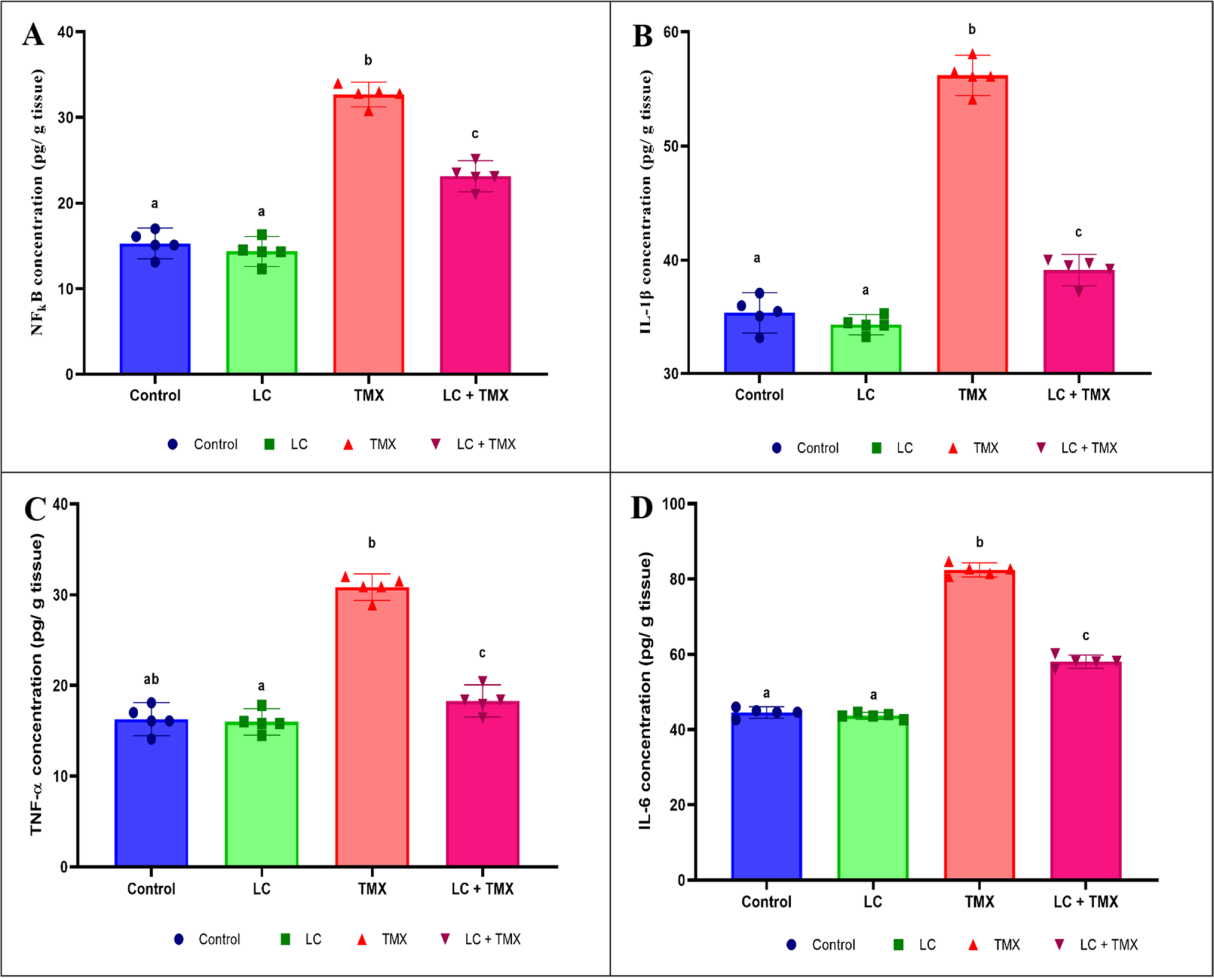
Effect of l-carnitine (LC) on the levels of (**A**) nuclear factor kappa B (NF-κB), (**B**) interleukin-1β (IL-1β), (**C**) tumor necrosis factor-α (TNF-α), and (**D**) interleukin-6 (IL-6) in the brain of rats treated with thiamethoxam (TMX). Values are presented as mean ± S.E.; *n* = 5 animals; different superscripts on the columns are significantly different at *P <* 0.05. a, *P* < 0.05 vs. control. b, *P* < 0.05 vs. LC. c, *P* < 0.05 vs. TMX. d, *P* < 0.05 vs. LC + TMX

TMX alone significantly elevated NF-κB (*F* (3, 16) = 190.47; *P* < 0.0001; 128%), IL-1β (*F* (3, 16) = 354.45; *P* < 0.0001; 64%), TNF-α (*F* (3, 16) = 142.93; *P* < 0.0001; 93%), and IL-6 (*F* (3, 16) = 1.03; *P* < 0.0001; 89%) levels in contrast to the control. In contrast, rats treated with LC had significantly lower levels of these proinflammatory cytokines (−29, −30, −41, and −30%, respectively) in the brain than the TMX-treated group (Fig. 4A–D). Two-way ANOVA revealed a significant interactive effect of LC and TMX on NF-κB (*F* = 4.793; *P* = 0.0001), IL-1β (*F* = 2.338; *P* = 0.0001), TNF-α (*F* = 4.738; *P* = 0.0001), and IL-6 (*F* = 4.121; *P* = 0.0001).

### Ameliorative effect of LC on the neurochemical disturbance caused by TMX

**Fig. 5:**
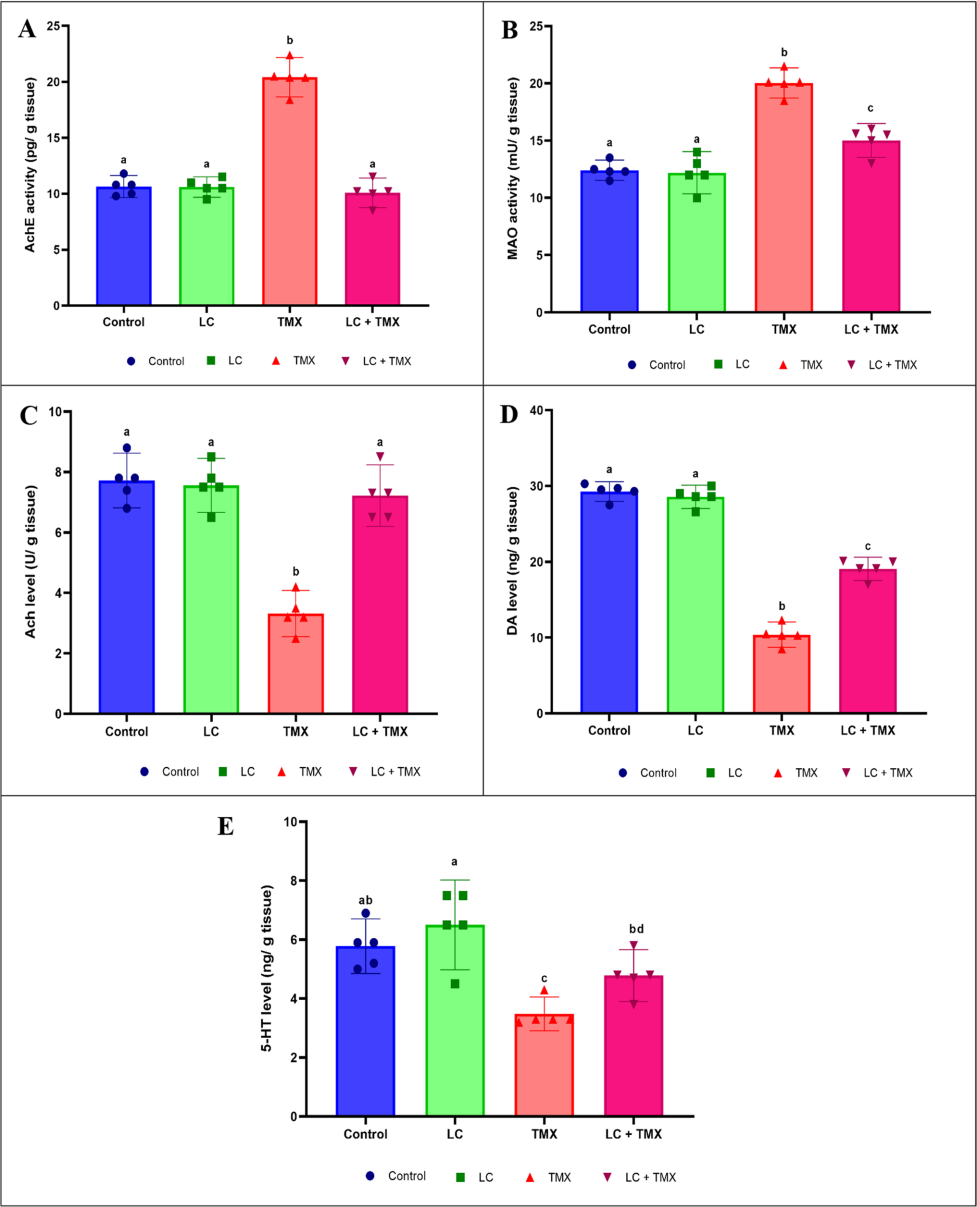
Effect of l-carnitine (LC) on the activities of (**A**) acetylcholinesterase (AchE) and (**B**) monoamine oxidase (MAO) as well as the levels of (**C**) acetylcholine (Ach), (**D**) dopamine (DA), and (**E**) serotonin (5-HT) in the brain of rats treated with thiamethoxam (TMX). Values are presented as mean ± S.E.; *n* = 5 animals; different superscripts on the columns are significantly different at *P <* 0.05. a, *P* < 0.05 vs. control. b, *P* < 0.05 vs. LC. c, *P* < 0.05 vs. TMX. d, *P* < 0.05 vs. LC + TMX

Figure 5A and B depicts the data on the brain AchE and MAO activity of rats treated with LC and/or TMX. TMX administration resulted in a significant (*P* ˂ 0.05 vs. controls) increase in AchE (*F* (3, 16) = 115.66, *P* < 0.0001; 93%) and MAO (*F* (3, 16) = 50.71, *P* < 0.0001; 64%) brain activity, the key indicators of nervous system function in the exposed rats. Furthermore, the reduction in Ach, DA, and 5-HT levels after TMX exposure was worsened in exposed animals (*F* (3, 16) = 42.04, *P* < 0.0001; −56%, *F* (3, 16) = 265.81, *P* < 0.0001; −64%, and *F* (3, 16) = 12.38, *P* < 0.0001; −46%, respectively) compared to control animals (Fig. 5C–E). In contrast to TMX-treated rats, the protective group treated with LC showed a significant decrease in AchE (−51%) and MAO (−25%) activity and a significant increase in Ach, DA, and 5-HT levels (117, 84, and 37%, respectively). Two-way ANOVA revealed a significant interactive effect of LC and TMX on AchE (*F* = 3.101; *P* = 0.0001) and MAO (*F* = 3.397; *P* = 0.0001) brain activity, and Ach (*F* = 1.588; *P* = 0.0001), DA (*F* = 6.344; *P* = 0.0001), and 5-HT (*F* = 761.262; *P* = 0.0001) levels.

### Ameliorative effect of LC on TMX-induced caspase-3 and GFAP activation

**Fig. 6:**
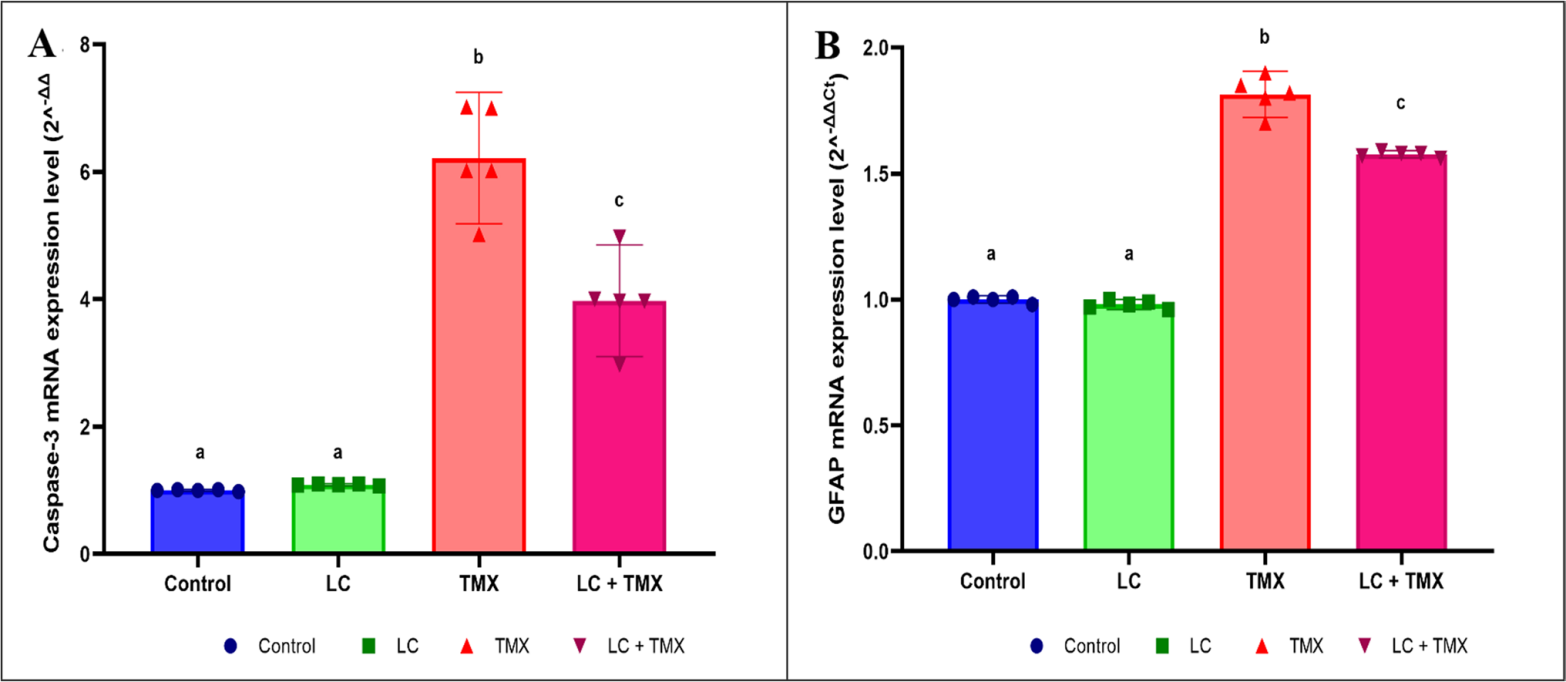
Effect of l-carnitine (LC) on the mRNA expression level of (**A**) caspase-3 and (**B**) glial fibrillary acidic protein (GFAP) in the brain of rats treated with thiamethoxam (TMX). The mRNA expression levels were normalized to the housekeeping gene (β-actin) and expressed as fold change (2^−ΔΔCT^). Values are presented as mean ± S.E.; *n* = 5 animals; different superscripts on the columns are significantly different at *P <* 0.05. a, *P* < 0.05 vs. control. b, *P* < 0.05 vs. LC. c, *P* < 0.05 vs. TMX. d, *P* < 0.05 vs. LC + TMX

The levels of caspase-3 and GFAP expression were measured in the experimental groups to investigate whether LC has anti-apoptotic and anti-astrogliosis effects in the brain. TMX-intoxicated rats had significantly higher expression levels of cleaved caspase-3 (*F* (3, 16) = 105.83; *P* < 0.0001; 471%) and GFAP (*F* (3, 16) = 582.97; *P* < 0.0001; 85%) than the control group. LC cotreatment with TMX, on the other hand, significantly (*P* ˂ 0.05 vs. TMX group) inhibited cleaved caspase-3 (−36%) and GFAP (−13%) expression in the brain (Fig. 6A, B). Two-way ANOVA revealed a significant interactive effect of LC and TMX on caspase-3 (*F* = 632.236; *P* = 0.0001) and GFAP (*F* = 2.399; *P* = 0.0001) expression levels.

## Discussion

The persistent use of chemical inputs such as pesticides has caused significant environmental damage as well as human illness. NEO intoxication has also been reported in humans, with clinical signs such as sleepiness, disorientation, dizziness, leukocytosis, muscular weakness, hypothermia, and convulsions (Hussain et al. 2022). With growing evidence of NEO neurotoxicity, there is an urgent need for a better understanding of their negative effects on nontarget organisms (Wang et al. 2018; Habotta et al. 2023). TMX is a potent NEO insecticide that has been shown to affect a variety of physiological indices and histological structures in albino rats (El-Din et al. 2023). LC has already been shown to have a significant neuroprotective effect against aspartame (Hamza et al. 2020), atrazine (Aziz et al. 2018), bisphenol A (Edres et al. 2018), and valproic acid (Nouri et al. 2022; Salimi et al. 2022) toxicity. Furthermore, LC has been shown to have significant antioxidant activity in animal models of depression (Martinotti et al. 2011), Alzheimer’s disease (Ahmed 2012), Parkinson’s disease (Salama and Elgohary 2021), neuropathic pain (Sarzi-Puttini et al. 2021), and epilepsy (Essawy et al. 2022). It was proven for the first time that administration of LC potently ameliorates brain oxidative stress, neuroinflammation, astrogliosis, and apoptosis after sequential 28-day TMX exposure in adult male rats.

Reactive oxygen species (ROS) produced from hazardous substances influence energy molecules such as proteins, lipids, and carbohydrates; the synthesis and utilization of these molecules may change under toxic stress (Kayis et al. 2019). Lipids are important in changing the structure and composition of cellular membranes and are also employed as energy sources during toxic biotransformation (Kayis et al. 2019). The lipid profile of TMX-exposed rats indicated a substantial increase in serum TC and TG, probably due to lipoprotein lipase hypoactivity, which breaks down triglycerides. Furthermore, LDL receptors become dysfunctional, increasing serum LDL-C, while inhibition of HDL-C concentration in the blood causes undesirable changes in lipid metabolism, producing hypercholesterolemia (Yousef et al. 2020). Increased levels of total lipids, cholesterol, and TG in this study could indicate high lipid peroxidation, which is associated with glycolipid metabolism disorders, loss of cell membrane integrity, and increased energy demand, resulting in cell damage, lipid accumulation, and apoptosis (Saad et al. 2022). Hyperstimulation of the nervous system causes energy demands, which activate hormone-sensitive triglyceride lipase in tissue, resulting in hydrolysis of stored triglycerides from fat stores and mobilization of free fatty acids in the bloodstream, resulting in increased serum total lipid concentration (Pothu et al. 2019). These results agreed with those of Wilkens et al. (2019) who found an increase in TG and very low-density lipoprotein (VLDL) levels in the plasma of bullfrog tadpoles exposed to two herbicides (sulfentrazone and glyphosate).

LC is required for long-chain fatty acid transport metabolism in mitochondria and consequently for energy metabolism. As acyl-carnitine derivatives, fatty acids pass through mitochondrial membranes and enter pathways for oxidation, acylation, chain shortening, or chain elongation-desaturation (Cha 2008). As a result, LC-dependent fatty acid transfer is critical to lipid metabolism; dietary supplementation of LC enhances fat utilization, resulting in a significant drop in plasma TG levels (Amin and Nagy 2009). The hypotriglyceridemic effect of LC may be due to its effect on lipase hyperactivity and antioxidant efficacy, which could lower serum lipid levels. Our findings are consistent with those of González-Ortiz et al. (2008) and Salama et al. (2012), who found that oral LC decreases blood TG, VLDL, TC, and LDL-C; improves dyslipidemia; and inhibits oxidative stress, as well as lowers cardiac parameters. LC treatment in obese rats considerably reduces serum hypertriglyceridemia via decreased triglyceride synthesis by the liver or suppression of triglyceride release from the liver (Rajasekar and Anuradha 2007).

NEOs exert their neurotoxic insecticidal action by binding to and activating nAchRs on the postsynaptic membrane of nerve cells (Duzguner and Erdogan 2012). They imitate the effect of Ach by opening ion channels, allowing cations such as Na^+^ and Ca^+2^ to enter and cause excitatory neurotransmission in the central nervous system (Rose 2012). The current results showed that the levels of those cations were dramatically reduced in TMX-treated rats; however, treatment with LC ameliorated these levels. The following mechanisms may be proposed based on the findings of these diverse studies. A first hypothesis is that by attaching this insecticide to the agonist binding site, the nAChRs conformation is altered, blocking the open channel of the receptor and thus reducing the influence of Na^+^ and Ca^+2^ ions on the nerve cell. A second hypothesis is that this pesticide reduces the conductance of open channels by binding to a site within the channel pore, preventing Na^+^ and Ca^+2^ ions from entering the nerve cell. A third hypothesis is that after this pesticide binds, different nAChRs subunits are activated, resulting in varying channel conductance levels (Akbas et al. 2014). Further investigations of channel characteristics, particularly the dose and voltage dependency of TMX effects, are required to confirm whether these hypotheses are correct. Another hypothesis is that NEOs may cause endocrine disruption in vertebrates (Habotta et al. 2021). Moreover, hyperglycemia and hyperlipemia in rats moved water out of the cells via hyperosmolarity and, as a result, dilutional hyponatremia (lower blood sodium level) (Khattab et al. 2020).

The administration of LC with antioxidant and/or free radical scavenging properties resulted in a considerable improvement in serum ionic electrolyte levels via renal glomeruli regeneration, which improved the kidney filtration process (Alabi et al. 2018). Furthermore, acetyl l-carnitine (ALC) inhibits lipofuscin formation and modulates membrane fluidity, potentially treating hyperglycemia and insulin insufficiency (Masoumi-Ardakani et al. 2020).

Oxidative stress caused by the overproduction of ROS and reactive nitrogen species, as well as changes in antioxidant enzyme activities, is implicated in NEO-induced injury to cellular molecules such as lipids, DNA, and proteins (Wang et al. 2018; Abdel-Razik et al. 2022). In the present study, brain oxidative damage was induced in TMX-exposed rats, as demonstrated by high levels of TBARS and significant inhibition of antioxidant enzymes. Similar findings of TMX-induced oxidative damage and free radical induction in male rat tissues have been reported (Habotta et al. 2021; Abd-Allah et al. 2022). Reduced levels of GSH were observed in this study due to its use in the conjugation process and/or its antioxidant activity in scavenging free radical products (Katić et al. 2021). Furthermore, the decrease in SOD and CAT activity suggests that TMX may considerably deplete the endogenous antioxidant system in the metabolic degradation of superoxide radicals and H_2_O_2_ due to increased oxidative stress (Katić et al. 2021; Habotta et al. 2021).

LC, a quaternary amine with strong antioxidant properties, has been employed to treat toxin-induced tissue damage (Edres et al. 2018). Because of its excellent free radical scavenging activity, its possible neuroprotective function against the progression of neurodegenerative diseases has gained much attention (Salama and Elgohary 2021). In our study, coadministration of LC and TMX significantly enhanced antioxidant status in the brain. The results are similar to previous findings that dietary supplementation with LC reduced malondialdehyde levels while increasing antioxidant enzymes such as CAT, SOD, and GSH (Li et al. 2014; Salama and Elgohary 2021). Aziz et al. (2018) found that LC decreases oxidative stress by interfering with arachidonic acid incorporation into phospholipids and the nicotinamide adenine dinucleotide phosphate (NADPH) oxidase pathway mediated by protein kinase C. It is also hypothesized that LC has antioxidant properties by removing harmful acetyl-CoA from the intracellular environment. Four alternative mechanisms might be used to explain how LC reduced TMX-induced brain injury: (1) LC directly removed ROS, suppressed brain cell lipid peroxidation, protected the cell membrane from oxidative stress, and maintained normal cell structure and functions; (2) LC indirectly scavenged free radicals by activating antioxidant enzyme systems in brain tissues to alleviate TMX-induced oxidative injury; (3) LC improved energy metabolism by suppressing the release of free electrons from the mitochondrial electron transport system, a prerequisite reaction for the generation of free radicals (Zaitone et al. 2012); and (4) LC chelated with metal ions by forming complexes with lysine and methionine amino acids, which are precursors for the first biosynthetic step of LC, while metal ions directly affect LC transfer via inhibition of carnitine acetyltransferase enzymatic activity (mitochondria) or indirectly by mediating organic cation transporter 2 activity (gut absorption) (Tjale et al. 2022).

Neuroinflammation is primarily caused by astrocyte and microglial activation and proliferation, activation, and translocation of transcription factors such as NF-κB, and increased production of cytotoxic cytokines such as TNF-α and IL-1β (Afshin-Majd et al. 2017; Pajares et al. 2020). The overproduction of free radicals in the brains of rats may be linked to TMX-mediated neuroinflammation, which activates the NF-κB signaling pathway. NF-κB increases the expression of inducible nitric oxide synthase (iNOS), proinflammatory cytokines (TNF-α and IL-6), and oxidative stress indicators (Al-Brakati et al. 2021). Habotta et al. (2023) found a significant increase in inflammatory cytokine levels as well as mRNA expression of IL-6, IL-1β, TNF-α, iNOS, and NF-κB in rat brain tissue exposed to TMX.

Controlling the degree of inflammatory reactions after brain damage may be useful because it has been linked to poor outcomes. In the present study, rats treated with LC exhibited considerably lower levels of these proinflammatory cytokines in the brain than TMX-treated rats. Supporting these findings, it has been demonstrated that LC can reduce inflammatory processes caused by the neurotoxic effects of 6-hydroxydopamine (6-OHDA), aspartame, and potassium dichromate by lowering the levels of NF-κB, TNF-α, and IL-6 (Afshin-Majd et al. 2017; Di Stefano et al. 2019; Salama and Elgohary 2021).

Neurotransmission is disrupted by NEO insecticides; hence, the cholinergic neurotransmitter system is anticipated to be the most injured in mammals. This system is critical for cognitive function control, and its malfunction has been associated with the onset of a variety of neurodegenerative disorders (Hampel et al. 2018; Abdel-Razik et al. 2022). Based on our findings, it is reasonable to speculate that TMX (or its metabolites) functions as a nicotinic agonist, activating nAChRs and changing cholinergic transmission to restore normal activity (Khaldoun-Oularbi et al. 2017). The current findings are consistent with those of Abdel-Razik et al. (2022), who revealed a significant increase in plasma AchE as a result of TMX treatment. Monoamine concentrations were also observed to be significantly lower in thiacloprid-exposed embryos. These changes may suggest a nervous system functioning deficit. These altered functions caused by TMX may be due to the identified oxidative damage, and they are most likely linked to neuronal dysfunction, as previously suggested by Farag et al. (2022). According to Saied and Hassan (2014), BPA (endocrine disruptor) such as TMX can disrupt dopaminergic transmission by altering various processes such as DA synthesis, release, and turnover, as well as the expression of both DA transporters and receptors. Decreased DA levels in the brain may impair metabolic activities carried out by catechol-O-methyltransferase (COMT) and MAO (Meiser et al. 2013). Changes in MAO activity can lead to altered biogenic amine concentrations, which can help individuals overcome stress (Abd Elkader et al. 2021).

LC is a necessary cofactor in lipid metabolism and, as a result, in the production of cellular energy. It participates in fatty acid β-oxidation by promoting the transport of long-chain fatty acids across the mitochondrial membrane (Ahmed 2012). The LC-protective group alleviated the activities of AchE and MAO as well as neurotransmitter levels. These results are supported by El-Sherbini et al. (2017). In the brain, LC and ALC play important roles in cerebral bioenergetics and neuroprotection via a variety of mechanisms, including antioxidant properties, modulation and promotion of synaptic neurotransmission, particularly cholinergic neurotransmission, and their ability to enhance neuronal metabolism in mitochondria (Ahmed 2012). Astrocytes, the brain’s immune cells, may produce enormous amounts of ketone bodies, which are thought to provide nearby neurons with easily transportable substrates for energy synthesis. The rate-limiting phase in astrocyte ketogenesis is the LC absorption mechanism (Inazu and Matsumiya 2008). Carnitine and ALC are structurally similar to choline and Ach; it has also been demonstrated that ALC has agonistic effects on cholinergic receptors and is a strong inhibitor of AchE (Sarzi-Puttini et al. 2021). ALC treatment increases DA and 5-HT release and receptor activity while decreasing the 5-hydroxyindoleacetic acid (5-HIAA)/5-HT ratio, the latter representing lower 5-HT turnover (Sarzi-Puttini et al. 2021). This could be due to increased mitochondrial membrane integrity, which could improve MAO function (Alves et al. 2009). The modulating effect of LC on the studied monoamines could be attributed to its ability to prevent neuronal loss by inhibiting ROS-mediated reactions via the reduction of microglial activation and the related production of oxygen intermediates and inflammatory factors (Essawy et al. 2022).

Although apoptosis is necessary for the control of normal physiological function throughout life, it also leads to abnormal cell death in pesticide-induced dementia (Ibrahim et al. 2015). There are two major apoptotic pathways, which differ in how they begin: the extrinsic or death receptor pathway and the intrinsic or mitochondrial pathway (Michelle et al. 2010). The current results showed that TMX-intoxicated rats had considerably higher caspase-3 expression levels than the control group. Previous studies have shown that independent and combined treatments with imidacloprid and esfenvalerate boosted caspase-3 activity in a dose-dependent manner (Ibrahim et al. 2015). Similarly, Wu et al. (2015) observed dramatically elevated apoptotic markers caspase-1 and caspase-3 in bee brains treated with imidacloprid in a time- and dose-dependent manner.

GFAP is an astrocyte intermediate filament protein that is thought to be a specific marker of astrocyte activation and/or injury after exposure to neurotoxic factors or other neurologic diseases (Yang et al. 2022; Gust et al. 2023). The current findings revealed substantial overexpression of the GFAP gene in the TMX-intoxicated group. These findings are consistent with previous studies that found elevated GFAP expression to be a sensitive marker of neurotoxins such as imidacloprid and thiacloprid (Katić et al. 2021; Forner-Piquer et al. 2021; Abomosallam et al. 2023). These changes may indicate higher dopaminergic and serotonergic turnover in the brain (Abd-Elhakim et al. 2018). Furthermore, oxidative stress and free radical production are increased because astrocytes, which maintain the integrity of the blood˗brain barrier, are targets of oxidative damage (Carvajal-Flores et al. 2020; Fulton et al. 2021).

In our study, combining LC and TMX dramatically reduced caspase-3 and GFAP expression in the brain. In this study, some of the neuroprotective effects of LC were related to its antiapoptotic and anti-inflammatory properties. The antiapoptotic action of ALC has previously been described (Virmani et al. 2013, Afshin-Majd et al. 2017). Dundar et al. (2016) found that ALC protected against doxorubicin (DOX)-induced severe myocardial caspase-3 immunoreactivity. Sarkar et al. (2015) previously demonstrated that ALC could reduce GFAP immunoreactivity in rotenone-induced dopaminergic toxicity. Furthermore, ALC pretreatment reduced GFAP expression in the striatum, which may be connected to its anti-inflammatory action (Afshin-Majd et al. 2017).

## Conclusion

LC has a wide range of pharmacological actions, the majority of which influence the neurological system. Our findings show that LC can mitigate TMX-induced brain injury in rats by reducing oxidative stress, modulating neurotransmission, and decreasing inflammation, astrogliosis, and apoptosis. These data support the hypothesis that LC may have preventive properties for neurological diseases.

## Data Availability

The data will be made available on request.
